# The osteogenic or adipogenic lineage commitment of human mesenchymal stem cells is determined by protein kinase C delta

**DOI:** 10.1186/s12860-014-0042-4

**Published:** 2014-11-25

**Authors:** Sooho Lee, Hee-Yeon Cho, Hang Thi Thuy Bui, Dongchul Kang

**Affiliations:** Ilsong Institute of Life Science, Hallym University, Anyang, Gyeonggi-do 431-060 Republic of Korea; Department of Biomedical Gerontology, Graduate School of Hallym University, Chuncheon, Gangwon-do 200-702 Republic of Korea

**Keywords:** hBMSCs, Osteogenic differentiation, Signal transduction, PKCδ, AMPK

## Abstract

**Background:**

Mesenchymal stem cells (MSCs) have the potential to differentiate into specialized cell lineages such as osteoblasts and adipocytes *in vitro*. There exists a reciprocal relationship between osteogenic and adipogenic differentiation of MSCs that an osteogenic phenotype occurs at the expense of an adipogenic phenotype and vice versa, which in turn influence one another’s phenotype through negative feedback loops. Thus, it is important to understand what signaling molecules modulate the lineage commitment of MSCs. Protein kinase C (PKC) plays a central role in cellular signal transduction for mediating diverse biological functions, and dysregulation of PKC activity is involved in various metabolic diseases including cancer, diabetes, and heart disease. Although the role of individual PKC isoforms has been investigated in various fields, the potential role of PKC in bone metabolism is not completely understood. In this study, we investigated the potential role of PKCδ in osteogenic lineage commitment of human bone marrow-derived mesenchymal stem cells (hBMSCs).

**Results:**

We observed that expression and phosphorylation of PKCδ were increased during osteogenic differentiation of hBMSCs. Pharmacological inhibition and genetic ablation of PKCδ in hBMSCs resulted in a significant attenuation of osteogenic differentiation as evidenced by reduced ALP activity and ECM mineralization, as well as down-regulation of the expression of osteoblast-specific genes. These effects were also accompanied by induction of adipogenic differentiation and up-regulation of the expression of adipocyte-specific genes involved in lipid synthesis in osteogenic induction of hBMSCs. Additionally, the activation of AMPK, which is a key cellular energy sensor, induced osteogenesis of hBMSCs. However, the inhibition of AMPK activity by compound C did not affect the activation of PKCδ at all, indicating that there is no direct correlation between AMPK and PKCδ in osteogenesis of hBMSCs.

**Conclusions:**

These results suggest that PKCδ is a critical regulator for the balance between osteogenesis and adipogenesis of hBMSCs and thus has a potential novel therapeutic target for the treatment of metabolic bone diseases.

**Electronic supplementary material:**

The online version of this article (doi:10.1186/s12860-014-0042-4) contains supplementary material, which is available to authorized users.

## Background

Human mesenchymal stem cells (hMSCs), also known as adult multipotent stem cells, have been identified in the bone marrow and in various tissues such as adipose tissue, synovial tissue, periosteum, perichondrium and cartilage [1]. These cells have the capacity of self-renewal and the potential to differentiate into specialized cell lineages, including osteoblasts, adipocytes and chondrocytes under permissive conditions [2]. Importantly, the potential of these cells in cell-based regenerative therapies hold tremendous promise for the treatment of various diseases including osteogenesis imperfecta, cardiovascular disease, and neurological disease [3,4]. Hence, it is important to understand the regulatory mechanism responsible for their differentiation.

Mature osteoblasts, which terminally differentiate into osteocytes, play an essential role for the initiation of bone mineralization and formation, leading to increased bone regeneration rate. Mineralized bone matrix is considered to be a hallmark of the final phase of osteogenic differentiation [5]. These processes are tightly controlled by the expression of osteogenesis-related genes, including alkaline phosphatase (ALP), which is not restricted to osteogenic cells and is also expressed in other cell types including embryonic stem cells, runt-related transcription factor 2 (RUNX2), osteocalcin (OCN), and osterix (OSX) [6]. Bone formation is dependent on the recruitment of an adequate number of osteoblasts and their osteogenic activity [7]. However, the impaired bone formation, which is functionally associated with decreased osteoblastic bone-forming activity, contributes to the pathogenesis of metabolic bone diseases including osteoporosis, osteomalacia, and Paget’s disease [8]. Therefore, understanding the molecular mechanisms underlying bone formation has emerged as a potential therapeutic approach for the treatment of these diseases.

Protein kinase C (PKC) is a family of serine/threonine protein kinases that is known to be involved in a multitude of physiological processes such as cell proliferation, differentiation, apoptosis, and survival. The PKC family consists of at least 11 distinct isoforms in mammals. PKCs are classified into three groups depending on their structure and cofactor requirements: Ca^2+^/diacylglycerol (DAG)-dependent classical PKC (α, β1, β2 and γ), DAG-dependent novel PKC (δ, ε, η, θ and μ), and Ca^2+^/DAG-independent atypical PKC (λ/ι and ζ). The PKC activity is also tightly regulated by its association with protein complexes and its intracellular distribution [9-12]. Recently, several studies have reported that specific PKC isoforms are involved in embryonic bone formation and remodeling by affecting both osteoblast and osteoclast activity [13-16]. These findings indicate that PKC could be targeted to drive osteogenesis in hMSCs. However, the exact mechanism of osteogenic differentiation of hMSCs regulated by PKC is still not fully understood.

In addition, the AMPK signaling pathway, a master regulator of cellular energy homeostasis, is involved in bone metabolism. Activation of AMPK stimulates bone formation *in vitro*, while the lack of either α or β subunit of AMPK results in reduced bone mass in mice [17]. AMPK has been reported as an upstream kinase of PKCδ in various cell types [18,19]. Although these studies suggest the possibility that AMPK/PKCδ pathway could participate in the MSC differentiation, there are no reports available as yet of the interrelationship between AMPK and PKCδ during differentiation into osteogenic lineage.

The aim of our study was to determine the role of specific PKC isoform in osteogenic differentiation. In this study, we employed human bone marrow-derived mesenchymal stem cells (hBMSCs). We identified that unlike other PKC isoforms, PKCδ mRNA and protein levels steadily increased during osteogenic differentiation of hBMSCs and further examined the role of PKCδ in the regulation of their osteogenic differentiation. We found that both pharmacological and genetic inhibition of PKCδ impaired osteogenic differentiation of hBMSCs, including a decreased ALP activity and matrix mineralization, as well as the down-regulation of osteogenic marker gene expression. Interestingly, we found that activation of AMPK, similar to changes in PKCδ expression, induced osteogenesis of hBMSCs. However, there was no direct correlation between PKCδ and AMPK under our experimental condition. We further showed that the effect of PKCδ inhibition on hBMSC osteogenic differentiation was exerted through a positive regulation of adipogenic differentiation. Notably, both pharmacological and genetic inhibition of PKCδ in hBMSCs exhibited more adipogenic phenotype than their counterparts under osteogenic condition. Thus, these findings demonstrate the potential importance of PKCδ in directing hBMSC differentiation and provide a promising new avenue for the treatment of metabolic bone diseases.

## Methods

### Cell culture

All cell culture media and supplements were obtained from Gibco (Carlsbad, CA, USA), unless otherwise indicated. Human bone marrow-derived mesenchymal stem cells (hBMSCs) were purchased from ScienCell Research Laboratories (Cat. No. 7500; Carlsbad, CA, USA) and maintained in growth medium (GM) consisting of α-Minimum Essential Medium (α-MEM) supplemented with 16.5% fetal bovine serum (FBS) and antibiotics (100 units/mL penicillin and 100 μg/mL streptomycin) at 37°C in a humidified atmosphere of 5% CO_2_ and 95% air. Cells between passages 3 and 10 were used for all experiments. For lentivirus production, HEK293T cells were cultured in Dulbecco’s modified Eagle’s medium (DMEM) containing 10% FBS and antibiotics (100 units/mL penicillin and 100 μg/mL streptomycin) at 37°C in a humidified atmosphere of 5% CO_2_ and 95% air.

### Osteogenic induction and alizarin red S staining

For osteogenic differentiation, hBMSCs were plated at density of 3 × 10^5^ cells/well on 6-well plates or 1 × 10^4^ cells/well on 96-well plates. After 2 days of incubation at which 100% confluent, hBMSCs were cultured for an additional 14 days in osteogenic differentiation medium (ODM) consisting of α-MEM supplemented with 10% FBS, 100 nM dexamethasone (Sigma-Aldrich, St. Louis, MO, USA), 10 mM β-glycerophosphate (Sigma-Aldrich, St. Louis, MO, USA), 50 μM ascorbic-2-phosphate (Sigma-Aldrich, St. Louis, MO, USA), 100 units/mL penicillin and 100 μg/mL streptomycin and then treated with either vehicle (DMSO; Sigma-Aldrich, St. Louis, MO, USA), or rottlerin (Calbiochem, La Jolla, CA, USA), or compound C (Calbiochem, La Jolla, CA, USA), respectively. Fresh medium was changed twice per week. Osteogenic differentiation of hBMSCs was assessed by alizarin red S staining for the presence of calcium deposits. Briefly, the cells were washed twice with PBS (Sigma-Aldrich, St. Louis, MO, USA), fixed with 4% formaldehyde (Sigma-Aldrich, St. Louis, MO, USA) for 30 min at room temperature, rinsed with distilled water, and then stained with 2% (w/v) alizarin red S (Sigma-Aldrich, St. Louis, MO, USA) dissolved in distilled water (pH 4.2; adjusted with 10% ammonium hydroxide [Sigma-Aldrich, St. Louis, MO, USA]) for 45 min. Cells were then washed extensively with distilled water and examined for mineralization of extracellular matrix (ECM). After imaging, the dye was eluted with 10% (w/v) cetylpyridinium chloride monohydrate (Sigma-Aldrich, St. Louis, MO, USA) in 10 mM sodium phosphate (pH 7.0; Sigma-Aldrich, St. Louis, MO, USA) for 1 h at room temperature, and the absorbance was measured at 570 nm using a Multiskan™ GO microplate reader (Thermo Scientific, Waltham, MA, USA).

### Oil red O staining

The hBMSCs were cultured in ODM as described above for 14 days. Accumulation of lipid droplets in differentiated adipocytes from hBMSCs was assessed by oil red O staining. Briefly, cells were washed twice with PBS and fixed with 4% formaldehyde for 30 min at room temperature. After washing two times with PBS, cells were stained with 0.6% oil red O (Sigma-Aldrich, St. Louis, MO, USA) in isopropanol (Sigma-Aldrich, St. Louis, MO, USA) for 2 h at room temperature. The stain was then removed, and the cells were rinsed five times with distilled water. The stained lipid droplets were observed with an inverted phase-contrast microscope (Olympus, Tokyo, Japan).

### ALP activity assay

Cellular ALP activity as an early marker of osteogenic differentiation was assessed at day 7. Cells were washed twice with PBS and then lysed with protein lysis buffer containing 50 mM Tris–HCl pH 7.4 (Promega, Madison, WI, USA), 150 mM NaCl (Sigma-Aldrich, St. Louis, MO, USA), 1 mM EDTA (Sigma-Aldrich, St. Louis, MO, USA), and 1% NP-40 (Sigma-Aldrich, St. Louis, MO, USA). ALP activity was determined colorimetrically by incubating protein lysates with the substrate *p*-nitrophenyl phosphate (Sigma-Aldrich, St. Louis, MO, USA) in a 96-well plate at 37°C for 30 min. The absorbance was measured at 405 nm and normalized against the corresponding protein concentrations. The values were expressed as fold change relative to undifferentiated cells.

### RNA extraction and reverse transcription PCR (RT-PCR)

Total RNA was extracted from cells using Trizol reagent (Invitrogen, Carlsbad, CA, USA), and cDNA was reverse-transcribed from 2 μg of total RNA with the GoScript™ Reverse Transcription System (Promega, Madison, WI, USA) according to the manufacturer’s instructions. The primer sequences used for PCR are given in Additional file 1: Table S1. The PCR was performed as follows: one cycle of 3 min at 95°C; 35 cycles of denaturation at 95°C for 30 sec, annealing at 56°C for 30 sec and extension at 72°C for 45 sec; and then a final cycle of 5 min at 72°C. The PCR products were loaded onto 1% agarose gel containing ethidium bromide (Promega, Madison, WI, USA). The expression data were normalized to β-actin mRNA levels in each sample.

### Western blot analysis

Cells were washed twice with PBS and lysed in RIPA lysis buffer including 50 mM Tris–HCl pH 7.4, 150 mM NaCl, 1 mM EDTA, 1% NP-40, 0.1% SDS (USB, Cleveland, OH, USA), 0.5% sodium deoxycholate (Sigma-Aldrich, St. Louis, MO, USA), 1 mM PMSF (Sigma-Aldrich, St. Louis, MO, USA), protease inhibitor cocktail (Pierce Biotechnology, Rockford, IL, USA), and phosphatase inhibitors containing 10 mM sodium fluoride (Sigma-Aldrich, St. Louis, MO, USA), 2 mM sodium orthovanadate (Sigma-Aldrich, St. Louis, MO, USA), 10 mM sodium pyrophosphate (Sigma-Aldrich, St. Louis, MO, USA). Protein concentration in the supernatant was determined using the Bradford assay (Bio-Rad Laboratories, Hercules, CA, USA). Equal amounts of total proteins (25 μg) were separated on 10% SDS-PAGE and transferred onto Hybond-ECL nitrocellulose membranes (Amersham, Arlington Heights, IL, USA). The membranes were blocked with Tris-buffered saline-Tween 20 (TBS-T: 10 mM Tris–HCl pH 7.6, 150 mM NaCl, and 0.1% Tween 20 [USB, Cleveland, OH, USA]) containing 5% nonfat dry milk (Becton Dickson and Company, Sparks, MD, USA) for 1 h at room temperature and incubated overnight at 4°C with specific primary antibodies diluted in TBS-T. The membranes were washed three times with TBS-T and then incubated with the appropriate horseradish peroxidase (HRP)-conjugated secondary antibodies for 1 h at room temperature. The blots were visualized using ECL detection reagents (Advansta, Menlo Park, CA, USA) and exposed to photographic films (Agfa HealthCare NV, Mortsel, Belgium). The antibody combinations and dilutions are detailed in Additional file 2: Table S2–1 and Table S2–2.

### Lentivirus production and titration

Lentiviral shRNA expression vectors for non-targeting and human PKCδ (*PRKCD*) were purchased from Sigma-Aldrich (St. Louis, MO, USA). Lentivirus was produced by using the calcium phosphate transfection protocol and the viral titre was measured as described previously [20]. Briefly, HEK293T cells were seeded into 10-cm dishes at a density of 5 × 10^6^ cells/dish and incubated overnight until they reached approximately 80% confluence. The cells were transfected with 10 μg of the shRNA transfer vector, 7.5 μg of psPAX2 viral packaging plasmid and 2.5 μg of pMD2G viral envelop plasmid in a 10-cm dish. Viral supernatants were collected at 48 h after transfection and used for transduction of target cells in the presence of 8 μg/mL polybrene (hexadimethrine bromide; Sigma-Aldrich, St. Louis, MO, USA) for 24 h. Cells were cultured in the presence of 2 μg/mL puromycin (Sigma-Aldrich, St. Louis, MO, USA) to select shRNA-transduced cells for 3 days and then used for differentiation. The viral titre was determined by relative vector particle numbers based on virion RNA and calculated according to the following formula: relative vector particles/mL (VP/mL) = (C × D)/V, where C = number of RNA copies, D = dilution of vector preparation (including the dilution into the PCR), and V = volume in mL.

### Statistical analysis

All data were expressed as the mean ± S.E. Differences between groups were examined for statistical significance using Student’s *t*-test and analysis of variance (ANOVA). The difference was considered to be significant if *P* <0.05.

## Results

### Up-regulation of PKCδ during osteogenic differentiation in hBMSCs

**Figure 1:**
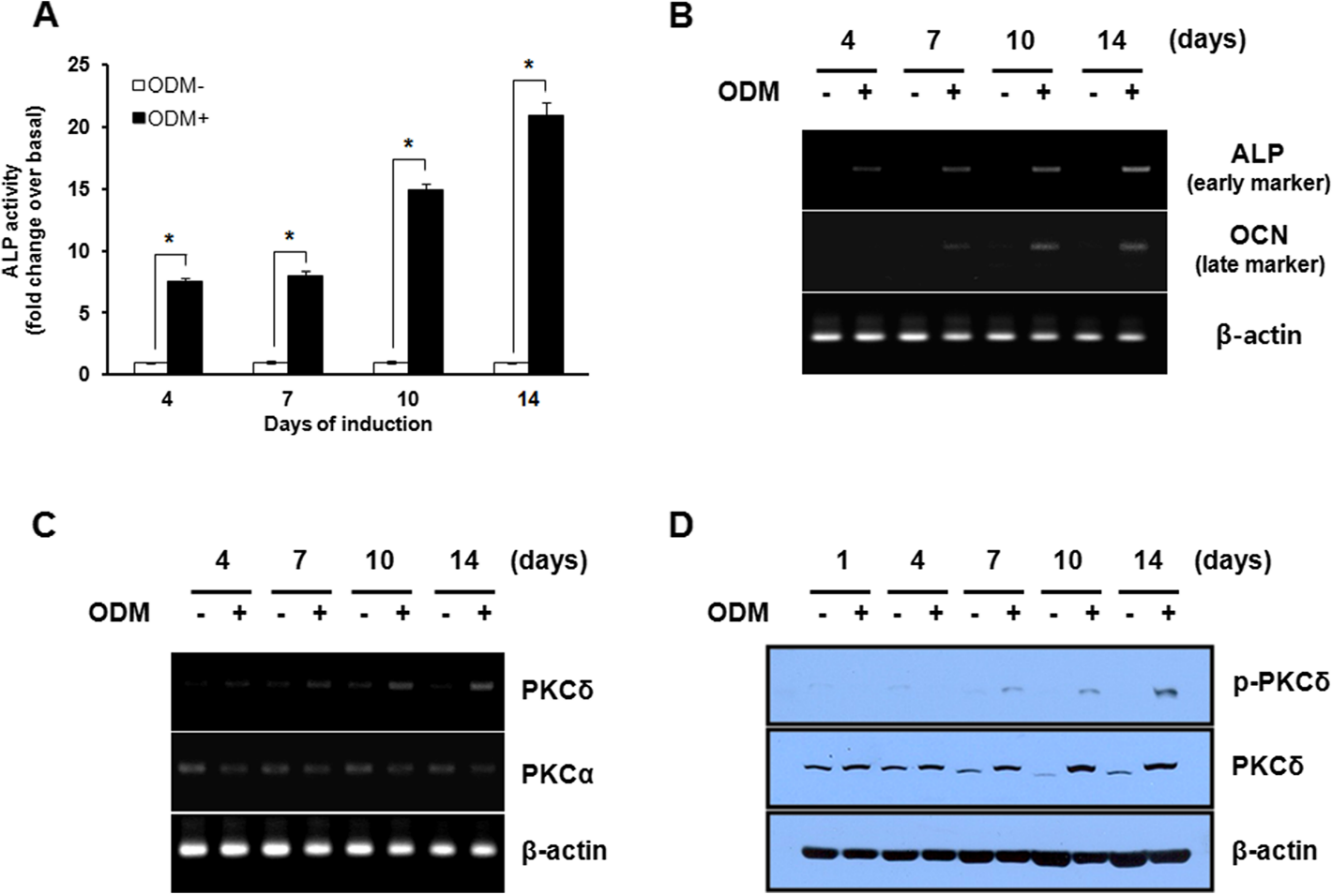
**Up-regulation of PKCδ during osteogenic differentiation in hBMSCs. (A-D)** Post-confluent hBMSCs were cultured in GM or ODM for the indicated times. The osteogenic differentiation potential of hBMSCs was estimated by ALP activity assay **(A)** and RT-PCR analysis of early and late osteogenic markers: ALP and OC **(B)** at the indicated times. **(C)** The mRNA expression of PKCα and δ were determined by RT-PCR at the indicated times after osteogenic induction of hBMSCs. **(D)** The protein level of PKCδ was determined by western blot analysis with the specified antibodies at the indicated times after osteogenic induction of hBMSCs. Data shown are means ± S.E. (*P <0.05 versus GM) of three independent experiments.

To examine the expression pattern of PKCδ during the osteogenesis of hBMSCs, we used an osteogenic differentiation model in which hBMSCs were incubated in either GM or ODM for 1, 4, 7, 10, and 14 days. Osteogenic differentiation potential of hBMSCs was confirmed by a significant increase in ALP activity and the mRNA levels of both early and late osteogenic markers, ALP and OCN, respectively (Figure 1A and B). This was associated with the mRNA and protein expressions of PKCδ and its phosphorylation, which was significantly up-regulated at 7, 10, and 14 days after the initiation of osteogenic induction (Figure 1C and D). Noticeably, the expression pattern of PKCδ is in stark contrast to that of PKCα under the same condition (Figure 1C), suggesting that among the PKC isoforms, PKCδ may act as a switch of osteogenic differentiation of hBMSCs. Taken together, these results indicate that osteogenic differentiation of hBMSCs is closely accompanied with expression and activation of PKCδ.

### Inhibition of PKCδ activity attenuates osteogenic differentiation in hBMSCs

**Figure 2:**
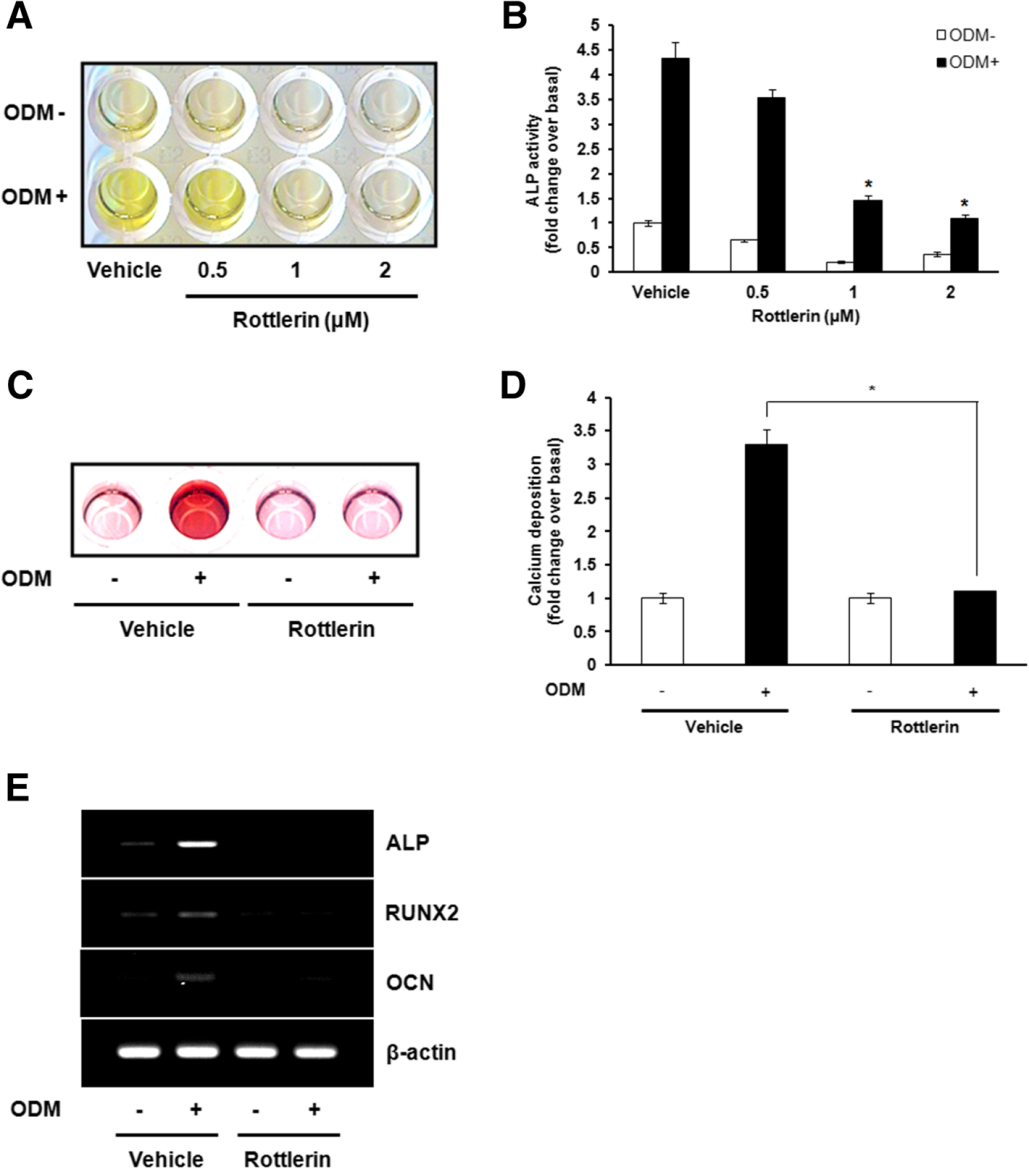
**Inhibition of PKCδ activity attenuates osteogenic differentiation in hBMSCs. (A)** Post-confluent hBMSCs were cultured in GM or ODM at the indicated concentrations of rottlerin, a specific PKCδ inhibitor. ALP activity was determined after osteogenic induction of hBMSCs for 7 days. **(B)** For quantitative determination of ALP activity, the absorbance at 405 nm of cell extracts was measured and expressed as the fold change of treated cells over vehicle-treated cells in GM. **(C)** Post-confluent hBMSCs were cultured in GM or ODM with 2 μM rottlerin. Alizarin red S staining was performed to monitor mineralization of hBMSCs after osteogenic induction for 14 days. **(D)** For quantitative determination, the absorbance of alizarin red S was measured at 570 nm and expressed as the fold change of treated cells over vehicle-treated cells in GM. **(E)** Post-confluent hBMSCs were cultured in GM or ODM with 2 μM rottlerin, and then harvested at 14 days. The mRNA expression of osteogenesis-related genes, including ALP, RUNX2, and OCN, was estimated by RT-PCR. The representative images from three independent experiments are shown. Data shown are means ± S.E. (*P <0.05 versus vehicle-treated cells in ODM) of three independent experiments.

To determine whether PKCδ activity is required for osteogenic differentiation of hBMSCs, we treated with various concentrations of rottlerin, a PKCδ-specific inhibitor, during osteogenic differentiation of hBMSCs. At 7 days after induction of differentiation, the intracellular ALP activity was evaluated by colorimetric assay. Treatment with rottlerin significantly decreased ALP activity in a dose-dependent manner (Figure 2A and B). After 14 days of induction, the mineralized matrix deposition was measured by alizarin red S staining. Treatment with 2 μM rottlerin completely blocked ECM mineralization (Figure 2C and D). To further confirm the effects of PKCδ inhibition on osteogenic differentiation, we analyzed the gene expression pattern of major osteogenic markers such as ALP, RUNX2, and OCN. As shown in Figure 2E, the mRNA levels of ALP, RUNX2, and OCN were significantly decreased by treatment with 2 μM rottlerin. These results suggest that PKCδ activation plays an important role in promoting osteogenic differentiation of hBMSCs.

### Knockdown of PKCδ inhibits osteogenic differentiation in hBMSCs

**Figure 3:**
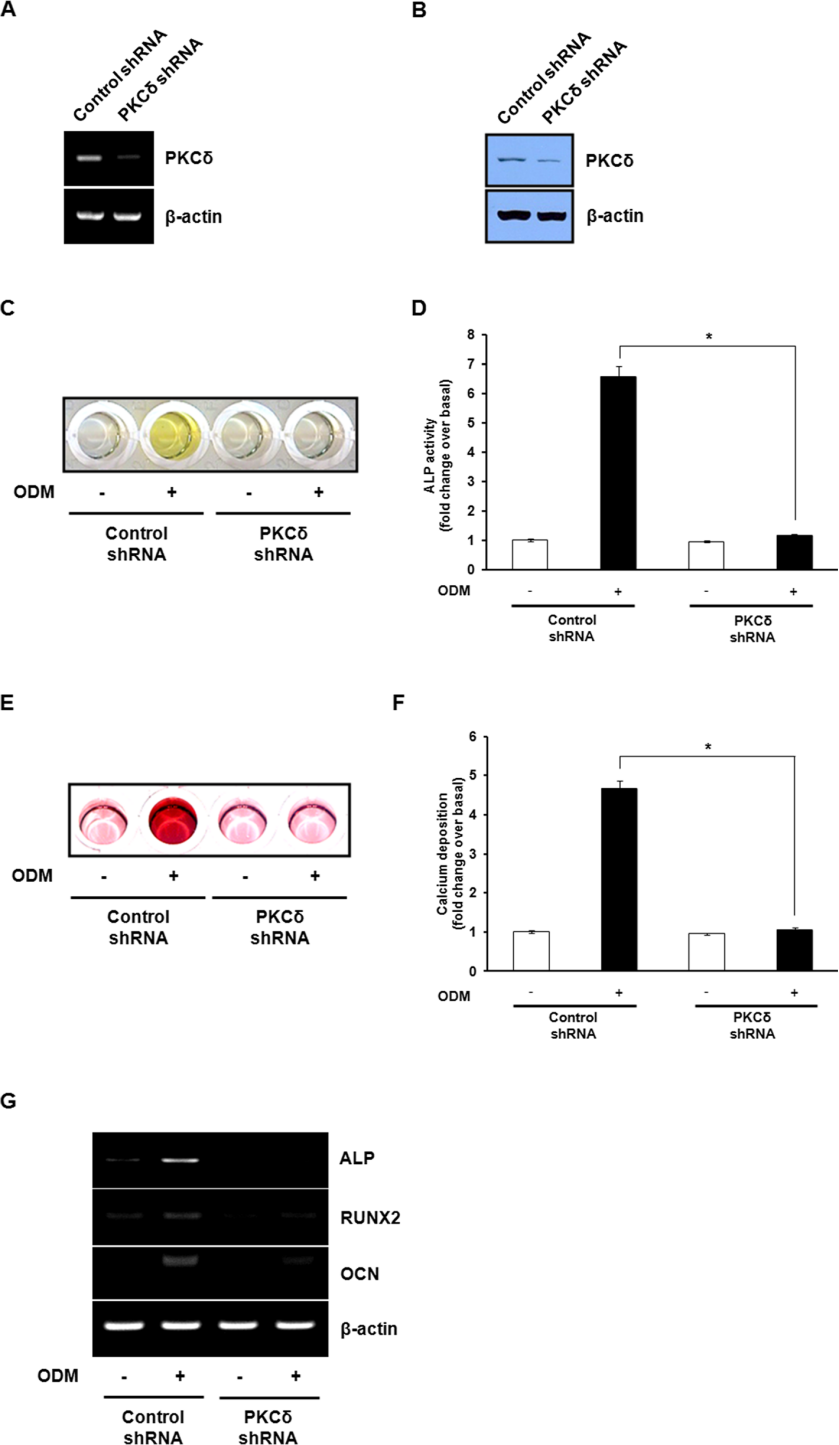
**Knockdown of PKCδ inhibits osteogenic differentiation in hBMSCs.** The knockdown efficacy of PKCδ shRNA was assessed by RT-PCR **(A)** and western blot analysis **(B)**. **(C)** Control shRNA- or PKCδ shRNA-transduced cells were cultured in GM or ODM. ALP activity assay was determined after osteogenic induction of hBMSCs for 7 days. **(D)** For quantitative determination, the absorbance at 405 nm of ALP reaction was measured and expressed as the fold change of cultured cells over control shRNA-transduced cells in GM. **(E)** Control shRNA- or PKCδ shRNA-transduced cells were cultured in GM or ODM, and differentiated osteoblasts were stained with alizarin red S. **(F)** For quantitative determination, the absorbance of alizarin red S was measured at 570 nm and expressed as the fold change of cultured cells over control shRNA-transduced cells in GM. **(G)** The mRNA expression of ALP, RUNX2, and OCN was estimated by RT-PCR. The representative images from three independent experiments are shown. Data shown are means ± S.E. (*P <0.05 versus control shRNA-transduced cells in ODM) of three independent experiments.

Although the direct inhibition of PKCδ activity by rottlerin has been demonstrated, a major problem has been its limited selectivity and undesired side effects [21]. To further determine the functional role of PKCδ in osteogenic differentiation, we applied lentivirus-mediated shRNA transduction to reduce PKCδ expression in hBMSCs. The knockdown efficacy of PKCδ shRNA was confirmed by RT-PCR and western blot analysis (Figure 3A and B). After 7 days of osteogenic differentiation, the ALP activity was significantly down-regulated in PKCδ shRNA-transduced cells compared with control shRNA-transduced cells (Figure 3C and D). We also observed that the mineralized matrix deposition was markedly suppressed in PKCδ shRNA-transduced cells after 14 days of osteogenic induction (Figure 3E and F). Additionally, the up-regulation of osteogenesis-specific genes during osteogenic differentiation was decreased in PKCδ shRNA-transduced cells (Figure 3G). Taken together, these results support the conclusion that PKCδ is necessary to trigger osteogenic differentiation of hBMSCs in a direct manner.

### AMPK activation is required for osteogenic differentiation of hBMSCs independently of PKCδ

**Figure 4:**
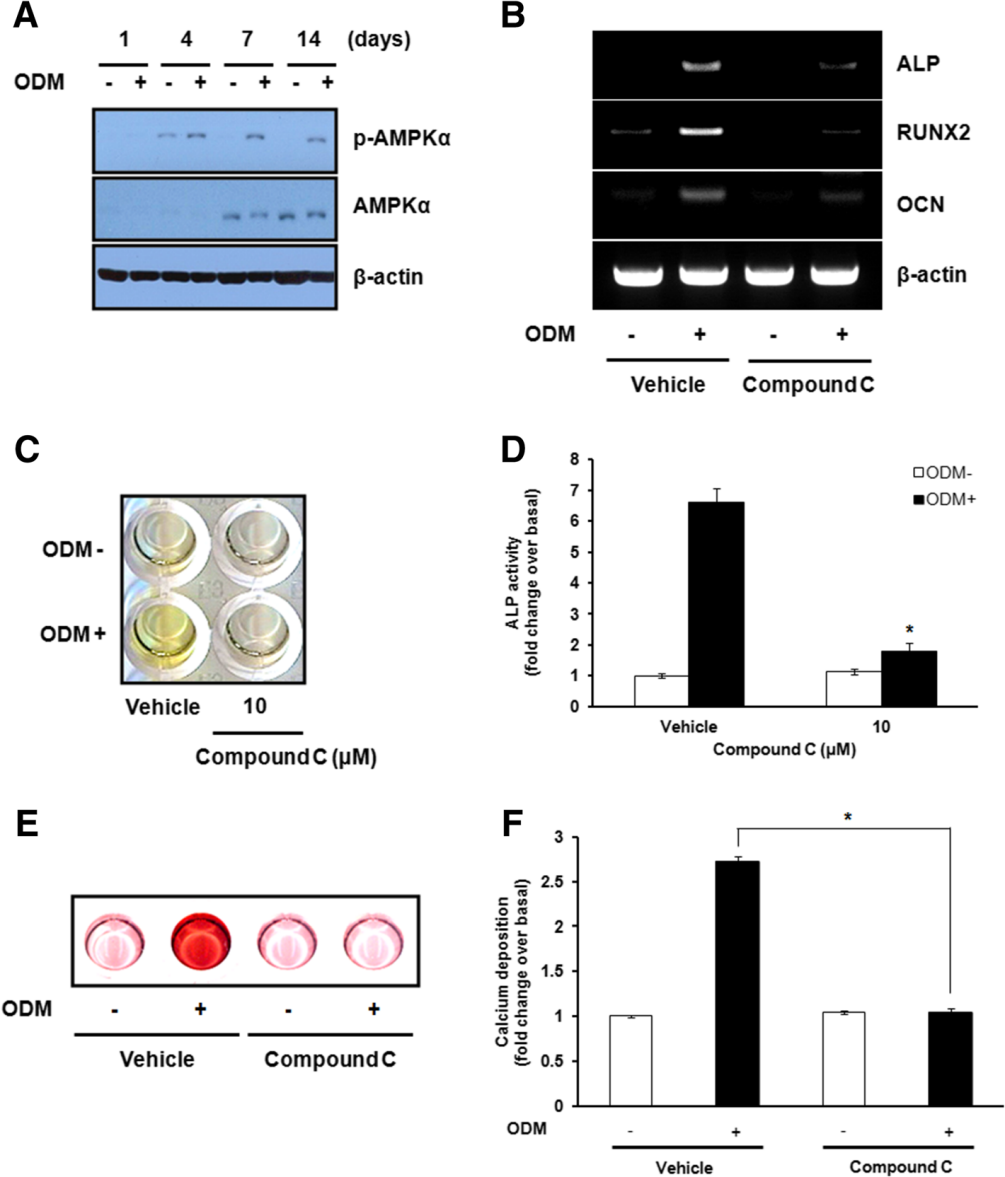
**AMPK activation is required for osteogenic differentiation in hBMSCs independently of PKCδ. (A)** Post-confluent hBMSCs were cultured in GM or ODM for the indicated times. Cell lysates were prepared and subjected to western blot analysis using the indicated antibodies. **(B)** Post-confluent hBMSCs were cultured in GM or ODM with 10 μM compound C, a specific inhibitor of AMPK, and then harvested after 14 days. The mRNA expression of ALP, RUNX2, and OCN was estimated by RT-PCR. **(C)** Post-confluent hBMSCs were cultured in GM or ODM with 10 μM compound C. After 7 days of osteogenic induction, ALP activity was determined by the ALP activity assay. **(D)** For quantitative determination, the absorbance at 405 nm of ALP reaction was measured and expressed as the fold change of treated cells over vehicle-treated cells in GM. **(E)** Post-confluent hBMSCs were cultured in GM or ODM with 10 μM compound C. Differentiated osteoblasts were stained with alizarin red S after 14 days of osteogenic induction. **(F)** For quantitative determination, the absorbance of alizarin red S was measured at 570 nm and expressed as the fold change of treated cells over vehicle-treated cells in GM. The representative images from three independent experiments are shown. Data shown are means ± S.E. (*P <0.05 versus vehicle-treated cells in ODM) of three independent experiments.

AMPK has been reported as an upstream kinase of PKCδ in various cell types [18,19]. Based on the previous observations, we investigated whether activation of AMPK might contribute to the osteogenic differentiation of hBMSCs, together with activation of PKCδ. The activation of AMPK was dramatically elevated on day 4 and maintained at a high level until day 14 after the initiation of osteogenic induction (Figure 4A). After 14 days of osteogenic induction, we observed that the mRNA levels of osteogenic differentiation markers, including ALP, RUNX2, and OCN, were completely impaired in the cells treated with 10 μM compound C, a specific inhibitor of AMPK (Figure 4B). Subsequently, we also observed that treatment with 10 μM compound C drastically reduced ALP activity (Figure 4C and D), which is consistent with the inhibitory effect of compound C on the formation of mineralized ECM (Figure 4E and F). Therefore, these results suggest that AMPK is potentially capable of stimulating osteogenic differentiation of hBMSCs. However, the inhibition of AMPK activity by compound C did not affect the activity of PKCδ at all, as assessed by western blot analysis, indicating that there is no direct correlation between AMPK and PKCδ in osteogenesis of hBMSCs (data not shown).

### Suppression of PKCδ-mediated osteogenic differentiation enhances the adipogenic phenotype of hBMSCs

**Figure 5:**
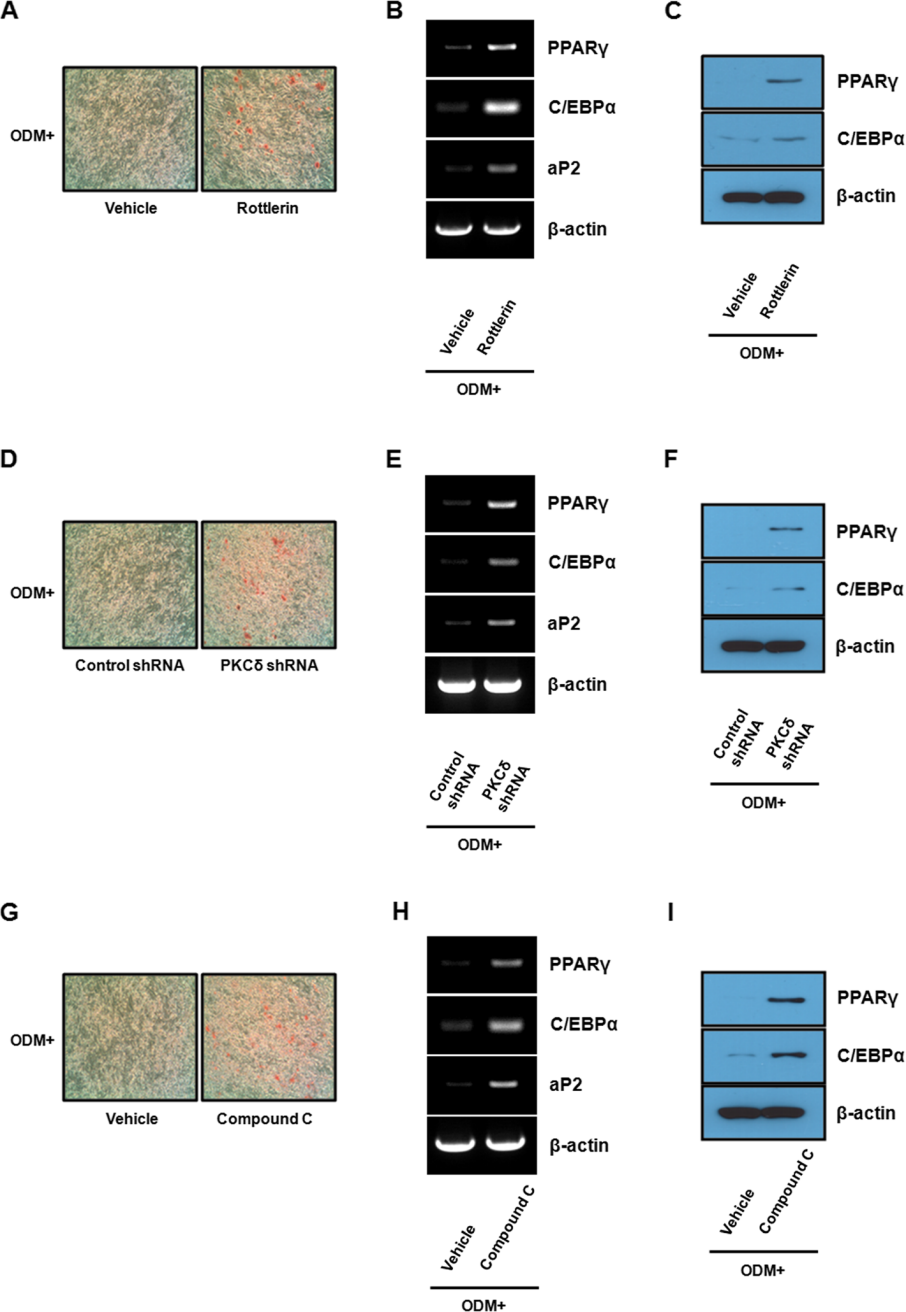
**Suppression of PKCδ-mediated osteogenic differentiation enhances the adipogenic phenotype of hBMSCs.** Post-confluent hBMSCs were cultured in ODM with 2 μM rottlerin **(A)** or 10 μM compound C **(G)**. **(D)** Control shRNA- or PKCδ shRNA-transduced cells were cultured in ODM. Differentiated adipocytes were stained with oil red O after 14 days of osteogenic induction and at magnification of x 100 photographed. **(B, E, and H)** After 14 days of osteogenic induction, RT-PCR was performed to estimate the mRNA expression of adipocyte-specific genes, including PPARγ, C/EBPα, and aP2, in the indicated groups. **(C, F, and I)** After 14 days of osteogenic induction, the protein expression levels of PPARγ and C/EBPα were determined by western blot analysis with the specified antibodies in the indicated groups. Data shown are representatives of three independent experiments.

The clinical and experimental implications have revealed an inverse relationship between osteogenic and adipogenic differentiation in bone marrow [22-25]. The inhibition of PKCδ in mouse preadipocytes leads to enhanced adipogenic differentiation by activating the expression of adipocyte-specific genes [26,27]. Therefore, we examined whether the effect of PKCδ inhibition on osteogenic differentiation causes induction of adipogenic differentiation of hBMSCs. Treatment with rottlerin induced accumulation of lipid droplets under osteogenic condition (Figure 5A). Similar to the lipid accumulation, RT-PCR results showed that the mRNA expression levels of all three adipogenic markers PPARγ, C/EBPα, and aP2 were increased in hBMSCs treated with rottlerin under osteogenic condition (Figure 5B). The protein expression levels of PPARγ and C/EBPα were also altered in the same manner as mRNA (Figure 5C). To further investigate the effect of PKCδ expression on adipogenic differentiation, we examined adipogenic differentiation potential of PKCδ-knockdown hBMSCs in osteogenic condition. Consistent with the effect of rottlerin that inhibition of PKCδ activity influences osteogenic differentiation by promoting PPARγ signaling, the lipid droplet accumulation and the expression of adipogenesis-specific markers at both the mRNA and protein levels were markedly elevated in PKCδ shRNA-transduced cells compared with control shRNA-transduced cells (Figure 5D, E, and F). Interestingly, identical to the effects of PKCδ inhibition, treatment with compound C increased the formation of lipid droplet and the expression of adipogenesis-specific genes at both the mRNA and protein levels under osteogenic condition (Figure 5G, H, and I). Collectively, these results strongly suggest that the effect of PKCδ inhibition on osteogenic differentiation could be sufficiently translated into adipogenic differentiation of hBMSCs.

## Discussion

PKC has been implicated in the regulation of a variety of cellular processes such as cell proliferation, differentiation, apoptosis, and survival. It was previously reported that modulation of PKC activity has several therapeutic effects in cancer and other metabolic diseases such as anti-tumorigenic properties, improved glucose metabolism and cardioprotective benefits [28]. However, the exact role of PKC in bone metabolism remains to be fully elucidated. In this study, we have identified the potential role of PKCδ as a key modulator of hBMSC differentiation. Our results indicate that the expression and phosphorylation of PKCδ were markedly elevated during osteogenic differentiation of hBMSCs, leading to a significant increase in ALP activity and matrix mineralization, as well as up-regulation of the expression of osteogenesis-specific genes. Moreover, inhibition of PKCδ not only inhibited osteogenic differentiation, but also promoted lipid accumulation in hBMSCs through increased expression of adipogenesis-specific marker genes under osteogenic condition. These findings suggest that the stimulatory effect of PKCδ on osteogenic differentiation of hBMSCs appears to have occurred, at least in part by suppression of adipogenesis signaling pathway. Taken all together, this is the first experimental study to support that PKCδ plays critical role in regulating osteogenic vs adipogenic differentiation of hBMSCs.

The expression pattern of PKC isofoms in different osteogenic precursor cell lines, including human MSCs and mouse osteoblastic MC3T3-E1 cells, has been previously investigated by several groups [29-31]. It has also been demonstrated that specific PKC isoforms have a role in regulating osteoblast activity *in vitro* (in cultured cells) and *in vivo* (in animal models). PKCα activation but not PKCβ suppressed osteogenic differentiation [14], whereas PKCδ promoted osteogenic differentiation through the transactivation of RUNX2 [32,33]. Moreover, the decreased bone formation during embryonic skeletal development has been shown in PKCδ knockout mice, probably due to delaying the onset of Osx expression [13]. We found that the increased expression of PKCδ is in contrast to the highly restricted expression of PKCα during osteogenic induction of hBMSCs (Figure 1). This is consistent with the previous findings that overexpression of PKCδ significantly decreased PKCα activity, while expression of dominant negative mutant of PKCδ significantly increased it *in vitro* [34]. The expression pattern of PKCδ positively correlates with the rapid induction of ALP acitivty and the up-regulation of early and late osteogenic marker genes, ALP and OCN, respectively. Therefore, these data suggest a potential role of PKCδ in the regulatory mechanism of osteogenic differentiation.

In the present study, suppression of PKCδ activity with a specific inhibitor, rottlerin, or depletion of PKCδ by lentiviral shRNA in hBMSCs inhibited induction of osteogenic differentiation (Figure 2 and Figure 3). We found that activation of PKCδ during osteogenic differentiation of hBMSCs leads to increased expression of RUNX2 and its downstream targets, ALP and OCN, which are known to be regulated by RUNX2. Moreover, inhibition of PKCδ using rottlerin or PKCδ shRNA completely reversed the osteogenic response of hBMSCs, suggesting that PKCδ functions as a potent activator of RUNX2 expression in bone development. Considerable evidence now suggests that PKCδ-dependent mechanism plays an important role in bone development. Several studies have indicated that osteogenic differentiation is associated with an increase in RUNX2 transcriptional activity through phosphorylation of RUNX2 at key residues by PKCδ, without changing the protein levels of RUNX2 [35,36]. The translocation of PKCδ from the cytoplasm to the nucleus in response to osteogenic condition could indeed influence the phosphorylation status and modulation of RUNX2 DNA-binding activity, which are concomitant with the enhanced OCN gene transcription [32,33,37]. Thus, although the functional interaction between PKCδ and RUNX2 was not assessed in the present study, it is possible that PKCδ could be directly responsible for enhancing osteogenic differentiation through both the regulation of RUNX2 expression and transcriptional activity in hBMSCs.

In terms of identifying the novel regulatory mechanisms that mediate the stimulatory effect of PKCδ on osteogenic differentiation of hBMSCs, it is worth noting that the effect of PKCδ on the osteogenic differentiation of hBMSCs occurs in parallel with an increased AMPK activity (Figure 4). Indeed, we found that AMPK activation increased markedly during osteogenic differentiation of hBMSCs and inhibition of AMPK reduced the gene expression of osteogenic markers in osteogenesis assays *in vitro*, including ALP activity and matrix mineralization. These results indicate an important role for AMPK in osteogenic differentiation of hBMSCs. A positive role of AMPK in driving and sustaining osteogenic differentiation is supported by the prior reports that AMPK activation facilitates bone formation by up-regulating expression of osteogenic lineage-specific genes [38,39]. Since both PKCδ and AMPK have a stimulating effect on osteogenic differentiation of hBMSCs, whether a direct connection between PKCδ and AMPK may synergize to accelerate osteogenic program of hBMSCs is a remaining question. Interestingly, AMPK was found to be associated with PKCδ activation in monocytic and lymphocytic cells [19]. In this study, although PKCδ and AMPK activation have identical effects on the regulation of hBMSC differentiation, no direct correlation was found between these two kinases. Consequently, PKCδ appears to coordinate osteogenic differentiation in hBMSCs independently of AMPK pathway. Further study is required to elucidate the novel upstream and downstream effectors of PKCδ during osteogenic differentiation of hBMSCs.

**Figure 6:**
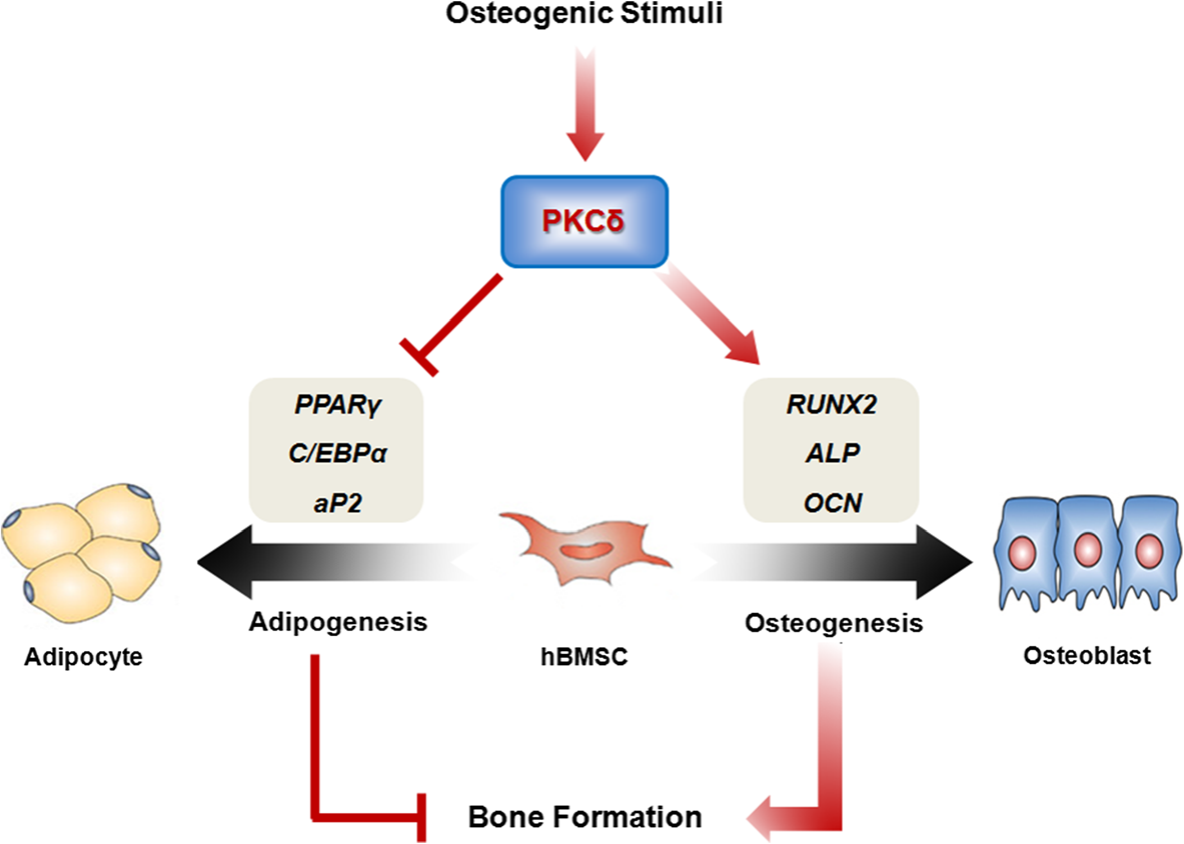
**PKCδ regulates bone formation in a direct and indirect manner.** The direct pathway is dependent on osteogenesis. The endogenous PKCδ in hBMSCs strongly stimulates the RUNX2 activity, thereby facilitating RUNX2-mediated osteogenic differentiation. The indirect pathway is independent on osteogenesis, which includes the potential negative regulation of adipogenic differentiation.

It is also noteworthy that inhibition of PKCδ using rottlerin or PKCδ shRNA suppressed osteogenesis but promoted adipogenesis of hBMSCs (Figure 5). These biphasic effects of PKCδ on hBMSC differentiation are likely to be partially explained by the inverse relationship between osteogenesis and adipogenesis in the bone marrow. It has been well established that the balance between osteogenesis and adipogenesis in MSCs depends on different signaling pathways that converge on the regulation of the two master transcription factors RUNX2 and PPARγ. The osteogenic and adipogenic signaling pathway may contribute to RUNX2 and PPARγ expression through a mutually negative interconnection [22-25]. Dysregulation of bone and fat formation is implicated with a high incidence of both osteoporosis and obesity [40,41]. We found PKCδ inhibition during osteogenic differentiation of hBMSCs resulted in a pronounced decrease in the expression of osteogenic transcription factor RUNX2 and up-regulation of major adipogenic transcription factors PPARγ and C/EBPα. These effects lead to suppression of bone formation and decrease of bone mineral content, and the formation of adipocyte-like phenotype containing intracellular lipid droplets in hBMSCs. Thus, it is plausible that PKCδ increases endogenous RUNX2 transcriptional activity, and up-regulated RUNX2 facilitates RUNX2-dependent gene transcription and osteogenesis, thereby repressing PPARγ-dependent gene transcription and adipogenesis (Figure 6). Meanwhile, it has been reported that the lineage commitment and differentiation of transformed haematopoietic progenitors is determined by the level of PKC activity, suggesting that the regulation of PKC activity is critical for governing the differentiation capacity of haematopoietic progenitor cells [42]. Taken together, these findings emphasize the important physiological effect of PKCδ on the relationship between bone and fat metabolism. Interestingly, identical to the effects of PKCδ inhibition, the adipogenic phenotype as a result of AMPK inhibition during *in vitro* osteogenesis of hBMSCs has also been observed (Figure 5). Our data are in agreement with the involvement of AMPK in stem cell differentiation [43], which supports evidence that AMPK may inhibit adipogenic differentiation by shifting stem cell fate toward osteogenic differentiation via a PKCδ-independent pathway.

## Conclusions

The results of present study demonstrate that PKCδ and AMPK has a crucial role in regulating the balance between osteogenesis and adipogenesis of hBMSCs. Since appropriate management of hBMSC differentiation is important for the development and maintenance of healthy bones, this study might provide a new insight into the regulatory mechanisms of hBMSC differentiation, further encouraging novel therapeutic strategies for improving bone regeneration.

## Additional files

Additional file 1: Table S1. List of all the primer sequences used for RT-PCR analysis. Additional file 2: Table S2–1. List of primary antibodies used for western blot analysis. **Table S2–2.** List of secondary antibodies used for western blot analysis.

## Abbreviations

MSCs: Mesenchymal stem cells
PKC: Protein kinase C
hBMSCs: Human bone marrow-derived mesenchymal stem cells
hMSCs: Human mesenchymal stem cells
ALP: Alkaline phosphatase
OCN: Osteocalcin
RUNX2: Runt-related transcription factor 2
OSX: Osterix
DAG: Diacylglycerol
GM: Growth medium
α-MEM: α-Minimum essential medium
FBS: Fetal bovine serum
DMEM: Dulbecco’s modified Eagle’s medium
ODM: Osteogenic differentiation medium
ECM: Extracellular matrix
PPARγ: Peroxisome proliferator-activated receptor γ
C/EBPα: CCAAT/enhancer-binding protein α
aP2: Adipocyte fatty acid binding protein
TBS-T: Tris-buffered saline-Tween 20
HRP: Horseradish peroxidase
ANOVA: Analysis of variance
