# Supplementary material for: The osteogenic or adipogenic lineage commitment of human mesenchymal stem cells is determined by protein kinase C delta
Source: BMC Cell Biol. 2014 Nov 25;15:42. doi: 10.1186/s12860-014-0042-4 (PMC4258059; doi:10.1186/s12860-014-0042-4)
Supplement: Additional file 1: Table S1. — List of all the primer sequences used for RT-PCR analysis. [file 12860_2014_42_MOESM1_ESM.pdf]

Table S1. List of all the primer sequences used for RT-PCR analysis

Gene	Accession Number	Forward/Reverse Primer (5' to 3')	Size
ALP	NM_000478.4	F- CAAAGGCTTCTTCTTGCTGG<br>R- AAGGGCTTCTTGTCTGTGTC	257 bp
RUNX2	NM_001024630.3	F- ACTGGGCCCTTTTTCAGA<br>R- GCGGAAGCATTCTGGAA	316 bp
OCN	NM_199173.4	F- ATGAGAGCCCTCACACTCCTC<br>R- GCCGTAGAAGCGCCGATAGGC	293 bp
PPARγ	NM_015869.4	F- GCTGTGCAGGAGATCACAGA<br>R- GGGCTCCATAAAGTCACCAA	205 bp
C/EBPα	NM_004364.4	F- AACCTTGTGCCTTGGAAATG<br>R- CCTGCTCCCCTCCTTCTCT	144 bp
aP2	NM_001442.2	F- AACCTTAGATGGGGGTGTCCTG<br>R- TCGTGGAAGTGACGCCTTTC	124 bp
PKCδ	NM_006254.3	F- ACCTCAAACTGGACAATGTG<br>R- GGTTGGTTCCCTTTCAAAGAG	346 bp
PKCα	NM_002737.2	F- CGAGGAAGGAAACATGGAAC<br>R- CCTTCCTGTCGGCAAGCATC	196 bp
β-actin	NM 001101.3	F- GGCATCGTGATGGACTCCG<br>R- GCTGGAAGGTGGACAGCGA	612 bp
