# Supplementary material for: The osteogenic or adipogenic lineage commitment of human mesenchymal stem cells is determined by protein kinase C delta
Source: BMC Cell Biol. 2014 Nov 25;15:42. doi: 10.1186/s12860-014-0042-4 (PMC4258059; doi:10.1186/s12860-014-0042-4)
Supplement: Additional file 2: Table S2–1. — List of primary antibodies used for western blot analysis. Table S2–2. List of secondary antibodies used for western blot analysis. [file 12860_2014_42_MOESM2_ESM.pdf]

Table S2-1. List of primary antibodies used for western blot analysis

Antigen	Host	Isotype	Species reactivity	Clonality	Supplier	Catalog number	Dilution
Phospho-PKCδ (Ser643)	Rabbit	IgG	H, M, R, Mk, X	Polyclonal	Cell Signaling Technology (Beverly, MA, USA)	#9376	1:1000
PKCδ	Rabbit	IgG	H, M, R, Mk, (X, B. Dg, Hr)	Monoclonal	Cell Signaling Technology (Beverly, MA, USA)	#9616	1:1000
Phospho-AMPKα (Thr172)	Rabbit	IgG	H, M, R, Mk, (B, C, Pg)	Polyclonal	Cell Signaling Technology (Beverly, MA, USA)	#2531	1:1000
AMPKα1/2	Rabbit	IgG	H, M, R	Polyclonal	Santa Cruz Biotechnology (Santa Cruz, CA, USA)	sc-25792	1:1000
PPARγ	Rabbit	IgG	H, M, R	Polyclonal	Santa Cruz Biotechnology (Santa Cruz, CA, USA)	sc-7196	1:1000
C/EBPα	Goat	IgG	H, M, R, X, Z	Polyclonal	Santa Cruz Biotechnology (Santa Cruz, CA, USA)	sc-9314	1:1000
β-actin	Mouse	IgG	H, M, R, C	Monoclonal	Santa Cruz Biotechnology (Santa Cruz, CA, USA)	sc-47778	1:2000

H, human; M, mouse; R, rat; Mk, monkey; X, xenopus; B, bovine; Dg, dog; Hr, horse; C, chicken; Pg, pig; Z, zebra fish

Table S2-2. List of secondary antibodies used for western blot analysis

Antigen	Host	Conjugate/Tag/Label	Supplier	Catalog number	Dilution
Rabbit IgG	Goat	HRP	Invitrogen (Carlsbad, CA, USA)	G21234	1:5000
Mouse IgG	Goat	HRP	Invitrogen (Carlsbad, CA, USA)	G21040	1:5000
Goat IgG	Bovine	HRP	Santa Cruz Biotechnology (Santa Cruz, CA, USA)	sc-2378	1:5000
